# TLR4 and TLR8 variability in Amazonian and West Indian manatee species from Brazil

**DOI:** 10.1590/1678-4685-GMB-2019-0252

**Published:** 2021-04-09

**Authors:** Tatiana Maia de Oliveira, Tibério Cesar Tortola Burlamaqui, André Luiz Alves de Sá, Breanna Breaux, Fábia de Oliveira Luna, Fernanda Löffler Niemeyer Attademo, Alex Garcia Cavalleiro de Macedo Klautau, Jairo Moura Oliveira, Leonardo Sena, Michael F. Criscitiello, Maria Paula Cruz Schneider

**Affiliations:** 1 Universidade Federal do Pará, Instituto de Ciências Biológicas, Belém, PA, Brazil.; 2 Universidade Federal Rural da Amazônia, Instituto Socioambiental e dos Recursos Hídricos, Laboratório de Genética Aplicada, Belém, PA, Brazil.; 3 Texas A&M University, College of Veterinary Medicine and Biomedical Sciences, Department of Veterinary Pathobiology, Comparative Immunogenetics Laboratory, College Station, TX, USA.; 4 Instituto Chico Mendes de Conservação da Biodiversidade (ICMBio), Centro Nacional de Pesquisa e Conservação de Mamíferos Aquáticos (CMA), Santos, SP, Brazil.; 5 Instituto Chico Mendes de Conservação da Biodiversidade (ICMBio), Centro Nacional de Pesquisa e Conservação de Mamíferos Aquáticos (CMA), Itamaracá, PE, Brazil.; 6 Centro de Estudos e Monitoramento Ambiental (CEMAM), Areia Branca, RN, Brazil.; 7 Instituto Chico Mendes de Conservação da Biodiversidade (ICMBio), Centro Nacional de Pesquisa e Conservação da Biodiversidade Marinha do Norte (CEPNOR), Belém, PA, Brazil.; 8 Universidade da Amazônia, Parque Zoológico da UNAMA (ZOOUNAMA), Santarém, PA, Brazil.; 9 Texas A&M University, Texas A&M Health Science Center, College of Medicine, Department of Microbial Pathogenesis and Immunology, College Station, TX, USA.

**Keywords:** Genetic diversity, Sirenian, Toll-like receptor, aquatic mammals

## Abstract

Amazonian (Trichechus inunguis) and West Indian (Trichechus manatus) manatees are aquatic mammals vulnerable to extinction found in the Amazon basin and the coastal western Atlantic. Toll-like receptors (TLR) play a key role in recognizing pathogen-associated molecular patterns using leucine-rich repeats (LRRs). We described the diversity of TLR4 and TLR8 genes in these two species of manatee. Amazonian manatee showed seven SNPs in TLR4 and the eight in TLR8, while West Indian manatee shared four and six of those SNPs, respectively. In our analysis, TLR4 showed one non-conservative amino acid replacement substitution in LRR7 and LRR8, on the other hand, TLR8 was less variable and showed only conserved amino acid substitutions. Selection analysis showed that only one TLR4 site was subjected to positive selection and none in TLR8. TLR4 in manatees did not show any evidence of convergent evolution compared to species of the cetacean lineage. Differences in TLR4 and TLR8 polymorphism may be related to distinct selection by pathogens, population reduction of West Indian manatees, or an expected consequence of population expansion in Amazonian manatees. Future studies combining pathogen association and TLR polymorphism may clarify possible roles of these genes and be used for conservation purposes of manatee species.

## Introduction

Sirenians are herbivorous aquatic mammals distributed in tropical and subtropical regions of the Americas, western coast of Africa, and Oceania (Husar, 1977; Domning, 1981; Bonde et al., 2012), evolutionarily related to elephants (*Loxodonta africana* and *Elephas maximus*) in the Superorder Afrotheria. The Sirenia Order is represented by the Indo-Pacific dugong (*Dugong dugon*) and three manatee species: the West Indian (*Trichechus manatus*), the Amazonian (*T. inunguis*) and the African (*T. senegalensis*) manatee (Husar, 1977; Domning, 1981; Marsh and Lefebvre, 1994). The Amazonian manatee is a freshwater species found in the Amazon basin, while the West Indian manatee consists of two subspecies: the Florida manatee (*T. m. latirostris*) is found on the coast of United States (Texas to Massachusetts), and the Antillean manatee (*T. m. manatus*), from the eastern Gulf of Mexico, Caribbean, Central and South America south to northeastern Brazil (Bonde *et al.,* 2012). An additional study using craniomorphometric characteristics and cytogenetics (Barros et al., 2016) indicates that West Indian manatees in the Brazilian coast must be a distinct species from the Antillean manatee. To complicate things further, evidence of hybrids of West Indian and Amazonian manatees reveals that those two species interbreed in transient habitats in the mouth of the Amazon river (Vilaça et al., 2019; Vilaça and Santos, 2020), with unknown consequences for adaptation and for the gene pool of the species involved.

All manatee species have a vulnerable conservation status according to IUCN (2019). Their decreasing numbers throughout their range is a result of past and present hunting for both meat and the leather trade (Domning, 1981), which might have affected genetic flow among populations, especially of West Indian manatees (Luna, 2013). However, the genetic diversity observed in some studies of manatees using neutral markers does not assess their ability to cope with environmental and anthropogenic changes (Garcia-Rodriguez et al., 1998; Vianna et al., 2006; Luna *et al.*, 2012). In fact, research on pathogens afflicting manatees has been conducted both in captivity and in natural environments in order to assess their health status (Bossart et al., 1998, 2002; Bando et al., 2014; Vélez et al., 2018), but only a few genetic studies have focused on genes related to the immune response (Breaux et al., 2017, 2018; Sá et al., 2019). Thus, poorly studied innate immune genes may provide insights not only on the health status of manatees (Gelain and Bonsembiante, 2019), but also on distinct selective pressures the manatee species may have undergone in distinct habitats.

A set of relatively conserved genes involved in the innate immune response against infectious agents is the Toll-like receptors (TLRs). TLR proteins are preferentially expressed on the cell surface or endogenous membrane compartments of specialized immune cells, such as dendritic cells, macrophages and neutrophils (Fleer and Krediet, 2007; Leulier and Lemaitre, 2008; Kawai and Akira, 2010; Cervantes et al., 2012; Novák, 2014). TLRs act as pattern recognition receptors (PRR) responsible for recognizing conserved structures of pathogens, called pathogen-associated molecular patterns (PAMP), inducing the production of cytokines to orchestrate limitation or removal of infectious agents such as bacteria, viruses, protozoa and fungi, signaling a series of events that lead to inflammatory and anti-viral responses (Janeway Jr., 1989; Fleer and Krediet, 2007; Leulier and Lemaitre, 2008; Kawai and Akira, 2010; Cervantes *et al.*, 2012; Novák, 2014; Medzhitov et al., 1997). The TLR molecule is structurally characterized by an ectodomain (ECD) containing leucine-rich repeats (LRRs) important for the recognition of PAMPs, a transmembrane domain (TM), and a cytoplasmic domain homologous to that of the interleukin-1 receptor, designated Toll/interleukin-1 receptor (TIR) domain, responsible for intracellular signaling (Fleer and Krediet, 2007; Leulier and Lemaitre, 2008; Kawai and Akira, 2010; Cervantes *et al.*, 2012; Novák, 2014).

Most genes directly involved with innate immunity are under strong purifying selection, which is expected based on their role as the first line of defense in recognizing conserved PAMPs of various pathogens (Mukherjee et al., 2009). However, several studies on TLR genes have demonstrated polymorphism in the vertebrates investigated, including birds, humans and other wild and domesticated mammalian species (Downing et al., 2010; Alcaide and Edwards, 2011; Areal et al., 2011; Grueber et al., 2012; Shen et al., 2012; Abrantes et al., 2013; Novák, 2014; Darfour-Oduro et al., 2015; Dalton et al., 2016a,b; Ishengoma and Agaba, 2017). The majority of the functional polymorphisms are at LRR amino acids (Werling et al., 2008) while the cytoplasmic TIR domain is more conserved, probably due to its role in intracellular signaling (Werling *et al.*, 2008).

As a first approach to study TLR in manatees we chose two functionally distinct molecules, TLR4 and TLR8. TLR4 is expressed on the cell surface and recognizes lipopolysaccharides (LPS) of primarily Gram negative bacteria (Akira et al., 2006). On the other hand, TLR8 is located on the endosomes and detects viral nucleic acids (Akira *et al.,* 2006; Barton, 2007; Yoneyama and Fujita, 2010). The aim of this study were to describe the diversity of *TLR4* and *TLR8* in Brazilian populations of *T. manatus* and *T. inunguis*.

## Material and Methods

### Samples

**Figure 1 -:**
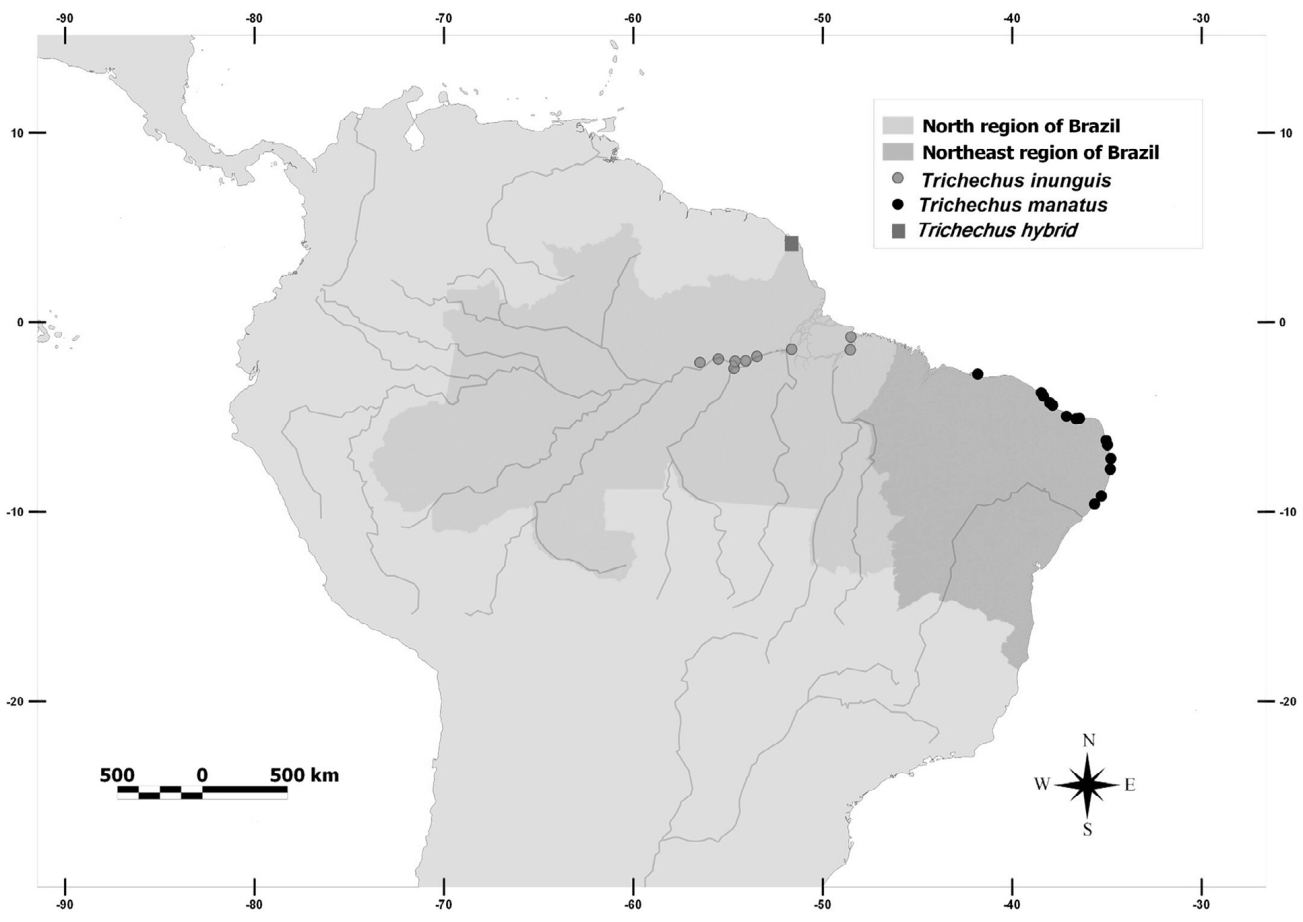
Map indicating the respective geographic collection sites of the manatees in North and Northeast regions of Brazil.

In this study we used 17 *T. manatus* of the National Center for Research and Conservation of Aquatic Mammals (CMA) of the Chico Mendes Institute for Biodiversity Conservation (ICMBio), Itamaracá, Pernambuco, Brazil, and 26 *T. inunguis* from the ZOOUNAMA (Santarém, Pará, Brazil), mostly born in the wild (Figure 1, detailed information on samples is in Table S1). All procedures were approved by the UFPA Ethics Committee under the permit CEUA/UFPA, CEPAE 68-2015. Blood sampling was performed by trained and authorized personnel under the license SISBIO 50641-2.

### TLR amplification and sequencing

DNA was extracted from peripheral blood leukocytes with the DNeasy Tissue & Blood kit (Qiagen; Hilden, Germany) following the manufacturer’s protocol. We used Primer-BLAST (https://blast.ncbi.nlm.nih.gov/Blast.cgi) and SerialCloner 2.6.1 (http://serialbasics.free.fr/) to design primers for the amplification of exon 1 of *TLR4* (2,242 bp) and the entire *TLR8* sequence (3,081 bp), which corresponds to a single exon, using the *T. manatus latirostris TLR4* (GI: 101353470) and *TLR8* (GI: 101348463) genes from the NCBI (National Center for Biotechnology Information) genomic database. Due to size restrictions for sequencing, we developed an amplification assay with overlapping amplicons, using multiple primer pairs (Table S2). Target exons were PCR amplified with the GoTaq®Flexi DNA Polymerase kit and GoTaq®Green Master Mix (Promega, Madison, USA), according to the manufacturer’s instructions. The PCR consisted of an initial denaturation step at 95 °C for 5 min, followed by 35 cycles of 95 °C for 1 min; optimum annealing temperature for each primer pair for 1 min (Table S2); 72 °C for 1 min, and final extension at 72 °C for 7 min.

The PCR products were Sanger sequenced (Applied Biosystems 3730 DNA Analyzer and 3500 Genetic Analyzer) in both directions, with the BigDye®XTerminator v3.1. kit (Applied Biosystems, Carlsbad, USA). Sequences were concatenated to form contigs for each sample; sequences were checked individually in Sequencer 4.1 (Gene Codes). Contigs were submitted to the National Center for Biotechnology Information (accession numbers are in Table S3). Sampled full length contigs were aligned using MAFFT online service (Katoh et al., 2017). Intron sequences were removed from our database. For the concatenated sequences, diploid genotypes were phased using Phase v2.1 implemented in DnaSP (Librado and Rozas, 2009), using 5,000 iterations with a burn-in of 500. Due to the size of amplicons, sequence quality was low in some individuals in the extremities of amplicons; in cases where low sequence quality hampered clear resolution of nucleotides in the overlapping region of amplicons, “Ns” were used in the alignments. Those “Ns” prevented a total overlapping for the TLR8 amplicons; thus, TLR8 sequences were analyzed as different segments - and we could only do phase analysis for each segment separately.

### TLR diversity and natural selection

We examined *TLR* sequences for evidence of selection using the HyPhy package46, implemented in the Datamonkey server (https://www.datamonkey.org; Delport et al., 2010) utilizing phased haplotypes obtained via DNAsp. We looked for evidence of positive selection using the mixed effects model of evolution (MEME), the more conservative single-likelihood ancestral counting (SLAC) method and unconstrained Bayesian aproximation for inferring selection (FUBAR) (Kosakovsky Pond and Frost, 2005; Murrell et al., 2012; Murrell *et al.*, 2013). MEME uses a mixed-effects maximum likelihood approach to determine nonsynonymous (d_N_) and synonymous (d_S_) substitution rates to detect episodic positive or diversifying selection at individual sites; SLAC calculates the expected and observed numbers of synonymous and nonsynonymous substitutions to infer selection and is a conservative test; and FUBAR is similar to SLAC but uses a Bayesian approach. We also analyzed other algorithms focusing on the gene locus. Thus, we compared Sirenians, Afrotherians and Artiodactyls, using BUSTED, which identifies genetic evidence of episodic positive selection, in which the rate of non-synonymous substitution is greater than the reason by the synonymous (Murrell *et al.*, 2015); RELAX, a framework hypothesis test that detects relaxed selection in a codon-based phylogenetic framework (Wertheim et al., 2014); and the aBSREL, a random effect branch-site model (Pond *et al.*, 2011; Smith et al., 2015). Accession numbers are provided in Table S4.

 Shen et al. (2012) estimated TLR4 sites under positive selection in the cetacean clade, another lineage of aquatic mammals not related to sirenians. In order to evaluate those two lineages of aquatic mammals, we compared those sites under positive selection in cetaceans to the homologues of sirenians. We also included in this analysis cattle and African elephant as related terrestrial mammals to cetaceans and manatees, respectively. This comparative analysis could not be performed for TLR8.

### TLR structure analysis

We used the amino acid sequences of the *T. m. latirostris* TLR4a and TLR4b isoforms (XP_004372178.2 and XP_012409812.1) and TLR8 (XP_004386403.1) from NCBI to identify the conserved domains by LRRfinder (Offord and Werling, 2012; http://www.lrrfinder.with/) and SMART (http://smart.embl-heidelberg.de/).

## Results

### Identification of TLR 4 and 8 polymorphisms

**Figure 2 -:**
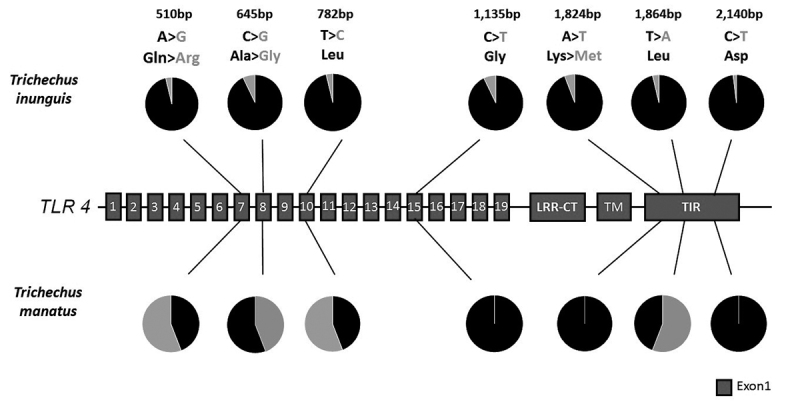
Identification of SNPs in the structure of the *TLR4* gene. In the graph the higher frequency of nucleotides is represented in black with synonymous and non-synonymous occurrences in two populations of manatees. The cartoon structure is represented by exon 1 (red). Rectangles represent LRRs (1-19), LRR-CT, trans-membrane (TM) and intracellular (TM) domains.

**Figure 3 -:**
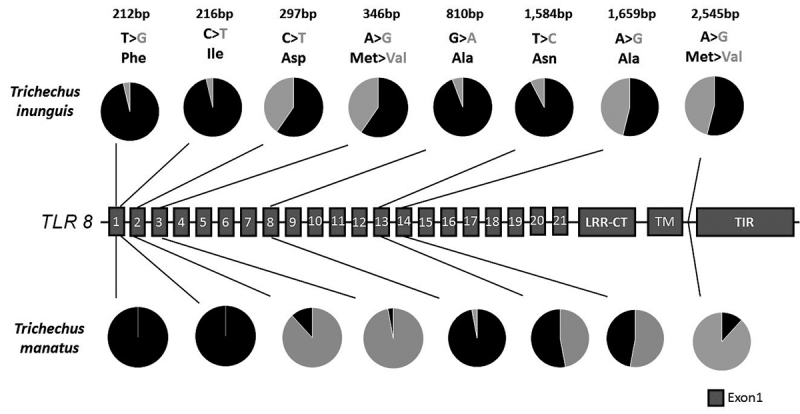
Identification of SNPs in the structure of the *TLR8* gene. In the graph the higher frequency of nucleotides is represented in black with synonymous and non-synonymous occurrences in two populations of manatees. The cartoon structure is represented by exon 1 (red). Rectangles represent LRRs (1-21), LRR-CT, trans-membrane (TM) and intracellular (TM) domains.

In the manatees, TLR4 and TLR8 had 20 and 22 LRRs, respectively. The frequency of the TLR4 SNPs varied between both species of manatees, but the number of SNPs were higher in Amazonian manatee in comparison to West Indian manatee for both TLR4 and TLR8, with no exclusive SNP for the latter species (Figures 2 and 3). Thus, both species shared four TLR4 SNPs: one synonymous substitution in LRR10 and one in TIR, one conservative non-synonymous substitution in LRR8 [Ala to Gly (nonpolar, hydrophobic)], and one non-conservative non-synonymous replacement in LRR7 [Glu (polar, hydrophilic, neutral) to Arg (polar, hydrophilic, basic)]. The remaining TLR4 SNPs were exclusive of the Amazonian manatee: one synonymous substitution in LRR15 and another in TIR, and one non-conservative non-synonymous replacement in TIR [Lys (polar, hydrophilic, basic) to Met (nonpolar hydrophobic)]. For TLR8, both species shared synonymous substitutions in LRR2, LRR8, LRR13 and LRR14, and conservative non-synonymous substitutions in LRR3, and between the TM and TIR domains with amino acid change from methionine to valine (nonpolar, hydrophobic) in both SNPs. In addition, Amazonian manatee showed two additional synonymous substitutions in LRR1.

The hybrid sample (Tman45) showed nucleotide sequences of TLR4 and TLR8 that are compatible with those found in West Indian manatee, i.e. it did not show any nucleotides that were exclusive for Amazonian manatee. Hence, we could not say whether this sample was a hybrid of the both manatee species studied based on TLR4 and TLR8 polymorphism (Table S5 and Table S6).

### Positive selection

Estimates for MEME and SLAC did not detect any site under selection for either TLR4 or TLR8 in our manatee samples. However, FUBAR analysis indicated one candidate site for positive selection (position 608 in our database) corresponding to the TIR domain of and three for negative selection (position 261, 621 and 713 in our database) in *T. inunguis* for TLR4, in the LRR10 and two in TIR region. The site under positive selection is equivalent to position 183 when compared to cetaceans (Shen et al., 2012; Table 1). In *T. manatus*, no site under positive selection was identified. In addition to the test for sites under selection, we tested whether the TLR sequences as a whole show evidence of selection by using the ABSREL, BUSTED and RELAX methods. RELAX was run with the sequences of Afrotherians and terrestrial Artiodactyls as the reference set to evaluate the selection intensification in the sequences of aquatic mammals. However, no evidence of positive selection was found for sirens, only RELAX suggested intensification in the selection in cetaceans for TLR8. Because there were few sites varying between both manatee species we compared them to the ones under positive selection in cetaceans according to Shen *et al.* (2012). Cetaceans corresponded to several species, while in manatee there were only two species. Our first approach was to compare TLR4 in West Indian manatee to its closest terrestrial relative, the African elephant. In this comparison, five positions revealed distinct amino acids (in bold in Table 1); then, we compared those five sites to the ones found in cetaceans and a close terrestrial relative, cattle, where we found that two of those sites had the same amino acids in cattle and manatee. Thus, from the three remaining sites in manatees that were different from African elephant, none of them had the same amino acids as the cetaceans studied by Shen *et al.* (2012). Thus, there was no evidence of convergent evolution in TLR4 between cetaceans and sirenians.

**Table 1 -:** Positive selection at amino acid sites of cetaceans according to Shen *et al.* (2012) in comparison to manatee, elephant, and cattle forTLR4 using FUBAR method. In bold, amino acids that are different between Florida manatee and African elephant.

AA position	Domain	Florida manatee	African Elephant	Cetaceans	Cattle
108	LRR6	His	His	Gln/Arg/His	His
128	LRR6	Gln	Gln	Glu/Pro	Glu
**150**	**Between LRR6-LRR7**	**Asn**	**Thr**	**His/Arg**	**His**
177	LRR7	Asn	Asn	Ile/Thr/Asn	Lys
179	LRR7	Asp	Asp	Lys/Glu/Gln	Gln
183	LRR7	Lys	Lys	Arg/Ser/Thr	Arg
207	LRR8	Ser	Ser	Lys/Thr/Arg	Gly
**208**	**LRR8**	**Asn**	**His**	**Asp/Ser**	**Asp**
221	Between LRR8-LRR9	Ala	Ala	Val/Ala/Met	Val
228	LRR9	Asp	Asp	Asn/Asp	Ser
**272**	**LRR11**	**Asp**	**Asn**	**His/Gly**	**Asp**
**278**	**LRR11**	**Asp**	**Glu**	**Glu/Asp/Lys**	**Glu**
308	LRR12	Thr	Thr	Thr/Ile/Ser	Thr
**324**	**LRR13**	**Gly**	**Asn**	**His/Asn/Ser/Lys**	**Gly**

## Discussion

Four out of seven SNPs found in the *TLR4* and six out of eight SNPs found in TLR8 of the Amazonian manatees were found in the West Indian manatee samples studied here. This higher level of variability in the Amazonian manatee genes could be related to distinct and more diversified pathogens found in fresh water habitats (Lang et al., 2009), or be another indication of population expansion signatures of this species as evidenced in other studies (Vianna et al., 2006), or may suggest that West Indian manatees have lost part of their variability due to recent decrease in population numbers, that could have affected at least some TLR genes, which could have conservation implications for both species.

The hybrid specimen analyzed here could not be differentiated from a West Indian manatee. This hybrid individual was studied by Luna (2013) and, although it had morphological characteristics of a West Indian manatee, its mitochondrial DNA is of an Amazonian manatee, while its karyotype had an intermediate number of chromosomes (n=50) between West Indian (n=48) and Amazonian (n-56) manatee. Although no conclusion may be drawn from only one hybrid individual, it reminds us of the importance of studying hybridization in manatees (Vilaça et al., 2019; Vilaça and Santos, 2020) and to evaluate the relative contribution of TLR polymorphism on the adaptation of those hybrids. As has been evidenced in some studies, TLR genes are to a great extent subjected to purifying selection (Alcaide and Edwards, 2011; Areal et al., 2011; Shen et al., 2012; Ishengoma and Agaba, 2017). When analyzing the levels of TLR polymorphism in human and chimpanzee populations, contrasted with the variation in the broader primate lineage, the TLR evolutionary pattern indicates that more episodic events of pathoghen-driven evolution have acted on a large time scale than ongoing selection in shorter periods (Wlasiuk and Nachman, 2010). In our manatee samples, TLR4 showed one non-conservative amino acid replacement substitution in LRR7 and LRR8, although clear signals of positive selection were not observed except in a position correspondent to the TIR domain of TLR4. TLR8, on the other hand, was less variable and showed only conserved amino acid substitutions. This pattern of higher variability in TLR4 and lower in TLR8 was also observed for other mammals (Kloch et al., 2018) and birds (Alcaide and Edwards, 2011; Dalton et al., 2016a), which may be attributed to the distinct pathogens each TLR identifies (Uematsu and Akira 2006), with TLR8 recognizing PAMPs in virus that must be less subjected to selection associated pathogen-host interactions (Kloch *et al.*, 2018).

As stated before, only one site of TLR4 was subjected to positive selection and none in TLR8 in manatees. On the other hand, in the primate lineage, *TLR4* shows the highest values of positive selection among other TLR genes (Wlasiuk and Nachman, 2010; Kloch et al., 2018). In cetaceans, TLR4 also reveals several sites under positive selection (Areal et al., 2011; Shen et al., 2012; Ishengoma and Agaba, 2017). However in cetaceans several different species were compared in Shen *et al.* (2012), while in sirenians only two closely related species were analyzed in our study. Notwithstanding, the lack of convergent evolution in the TLR4 sites under positive selection in cetaceans in comparison to sirenians may be attributed not only to differences in the pathogens found in their environment (Shen *et al.*, 2012), but with other ecological variables, mainly the exclusive herbivory of manatees, which makes it less likely for them to come into contact with different pathogens and commensals found in cetacean preys (Ishengoma and Agaba, 2017). Thus, although several lineages of marine mammals show evidence of convergent evolution (Tenaillon et al., 2012; Stern, 2013; Foote et al., 2015; Chikina et al., 2016), it might be more difficult to find similarities in immune response between highly divergent groups of aquatic mammals.

In conclusion, West Indian and Amazonian manatees showed polymorphism in TLR4 and TLR8, with higher variability in the latter. It is unclear at this moment whether differences in polymorphism are related to distinct selection by pathogens, population reduction of West Indian manatees, or an expected consequence of population expansion in Amazonian manatees. Genes related to innate immune response, such as TLR, may be good candidates for screening to assess manatee health status and evaluate the importance of their hybridization zone, as well as their conservation status. Future studies combining pathogen association and TLR polymorphism may clarify possible roles of these genes and be used for conservation purposes of manatee species.

## Supplementary material

The following online material is available for this article:

Table S1 - Information of samples collected in two manatee species from Brazil.

Table S2 - Amplified fragments of each TLR.

Table S3 - Accession number of TLR4 and TLR8 sequences in Amazonian and West Indian manatees deposited in GenBank.

Table S4 - Accession numbers of the TLR4 and TLR8 from GenBank.

Table S5 - Identification of SNPs for the TLR4 in Amazonian (*Trichechus inunguis*) and West Indian (*Trichechus manatus*) manatees.

Table S6 - Identification of SNPs for the TLR8 in Amazonian (*Trichechus inunguis*) and West Indian (*Trichechus manatus*) manatees.
