# Supplementary material for: TLR4 and TLR8 variability in Amazonian and West Indian manatee species from Brazil
Source: Genet Mol Biol. 2021 Apr 9;44(2):e20190252. doi: 10.1590/1678-4685-GMB-2019-0252 (PMC8042642; doi:10.1590/1678-4685-GMB-2019-0252)
Supplement: Table S1 - [file 1415-4757-GMB-44-2-e20190252-s1.pdf]

Genetics and Molecular Biology
https://doi.org/10.1590/1678-4685-GMB-2019-0252

Supplementary Material to "TLR4 and TLR8 variability in Amazonian and West Indian manatee species from Brazil"

Table S1. Information of samples collected in two manatee species from Brazil.

Code	Species	Gender	City	State	Birth (wild or captivity)
Tinu01	*Trichechus inunguis*	Male	Curupá	Pará	Wild
Tinu02	*Trichechus inunguis*	Female	Salvaterra	Pará	Wild
Tinu03	*Trichechus inunguis*	Male	Alenquer-	Pará	Wild
Tinu04	*Trichechus inunguis*	Female	Monte Alegre	Pará	Wild
Tinu05	*Trichechus inunguis*	Male	Prainha	Pará	Wild
Tinu06	*Trichechus inunguis*	Female	Monte Alegre	Pará	Wild
Tinu08	*Trichechus inunguis*	Female	Santarém	Pará	Wild
Tinu09	*Trichechus inunguis*	Female	Alenquer	Pará	Wild
Tinu10	*Trichechus inunguis*	Male	Terra Santa-	Pará	Wild
Tinu11	*Trichechus inunguis*	Male	Santarém	Pará	Wild
Tinu12	*Trichechus inunguis*	Male	Óbidos	Pará	Wild
Tinu13	*Trichechus inunguis*	Female	Santarém	Pará	Wild
Tinu14	*Trichechus inunguis*	Male	Óbidos	Pará	Wild
Tinu15	*Trichechus inunguis*	Male		Pará	wild
Tinu16	*Trichechus inunguis*	Female	Ilha das Onças	Pará	wild
Tinu33	*Trichechus inunguis*	Male	Óbidos-	Pará	Wild
Tinu34	*Trichechus inunguis*	Female	Santarém	Pará	Wild
Tinu35	*Trichechus inunguis*	Female	Santarém	Pará	Wild
Tinu36	*Trichechus inunguis*	Female	Santarém	Pará	Wild
Tinu39	*Trichechus inunguis*	Female	Santarém	Pará	Wild
Tinu41	*Trichechus inunguis*	Male	Óbidos	Pará	Wild

Code	Species	Gender	City	State	Birth (wild or captivity)
Tinu42	*Trichechus inunguis*	Male	Santarém	Pará	Wild
Tinu43	*Trichechus inunguis*	Female	Óbidos	Pará	Wild
Tinu46	*Trichechus inunguis*	Female	Santarém	Pará	Wild
Tinu47	*Trichechus inunguis*	Male	Santarém	Pará	Wild
Tinu48	*Trichechus inunguis*	Female	Óbidos	Pará	Wild
Tman18	*Trichechus manatus*	Male	Beriberi-	Ceará	Wild
Tman19	*Trichechus manatus*	Male	Humberto dos Campos	Maranhão	Wild
Tman20	*Trichechus manatus*	Female	Pipa	Rio Grande do Norte	Wild
Tman21	*Trichechus manatus*	Male	Pr. Sagi	Rio Grande do Norte	Wild
Tman23	*Trichechus manatus*	Female	Praia do Oitero	Paraíba	Wild
Tman24	*Trichechus manatus*	Male	Praia do Diogo Lopes-Macau	Rio Grande do Norte	Wild
Tman25	*Trichechus manatus*	Female		Ceará	Wild
Tman26	*Trichechus manatus*	Female	Barra de Sucatinga	Ceará	Wild
Tman27	*Trichechus manatus*	Male	Aquiraz	Ceará	Wild
Tman28	*Trichechus manatus*	Female	Pr. Barro Preto	Ceará	Wild
Tman29	*Trichechus manatus*	Female	Praia de Pratagy	Alagoas	Wild
Tman30	*Trichechus manatus*	Female	Areia Branca	Rio Grande do Norte	Wild
Tman31	*Trichechus manatus*	Female	Itamaracá	Pernambuco	Captivity
Tman32	*Trichechus manatus*	Male	CMA	Ceará	Captivity
Tman37	*Trichechus manatus*	Female	Itamaracá	Pernambuco	Captivity
Tman44	*Trichechus manatus*	Female	Itamaracá	Pernambuco	Captivity
Tman45	*Trichechus manatus*	Male	Oiapoque	Amapá	Wild

Tman 45: Trichechus hybrid.
