# Supplementary material for: TLR4 and TLR8 variability in Amazonian and West Indian manatee species from Brazil
Source: Genet Mol Biol. 2021 Apr 9;44(2):e20190252. doi: 10.1590/1678-4685-GMB-2019-0252 (PMC8042642; doi:10.1590/1678-4685-GMB-2019-0252)
Supplement: Table S2 - [file 1415-4757-GMB-44-2-e20190252-s2.pdf]

Genetics and Molecular Biology
https://doi.org/10.1590/1678-4685-GMB-2019-0252

Supplementary Material to "TLR4 and TLR8 variability in Amazonian and West Indian manatee species from Brazil"

Table S2. Amplified fragments of each TLR. Combination of primers, optimum annealing temperature (TM) and expected size of the fragments.

Fragments	Primers	Sequences (5'-3')	TM (°C)	Size (bp)
TLR4-1	TLR4 F1	ATTTGCCCTCCTTGATATCTTTA	60	796
	TLR4 R1	CTGCAGAAACATTTGCCAAGC		
TLR4-2	TLR4 F2	TCTGCCCTGGAGGGACTATG	60	917
	TLR4 R2	GCTCGCAAACACAAGCAAAG		
TLR4-3	TLR4 F3	ACTCCCTTCAGGTTCTGGACT	60	905
	TLR4 R3	GTGGGAAAGTGTCACAGGAGTT		
TLR8-1	TLR8 F1	CATACGAGGTAGGGCGAAGC	60	877
	TLR8 R1	TTGAAACGCAAGGGGATGGAT		
TLR8-2	TLR8 F2	CAAAACTGCCTCGCTCCCTA	60	1039
	TLR8 R2	TAGACGGTGCGTTACCCCAG		
TLR8-3	TLR8 F3	GCTTTTGGTGACATCGCCTG	60	928
	TLR8 R3	GGCATCTGAAACGCAAGTTGT		
TLR8-4	TLR8 F4	AACGAAGACAGCCACCAACC	64	874
	TLR8 R4	CATGAGACAGAAGAGTAGTTGCTTTG		
