# Supplementary material for: TLR4 and TLR8 variability in Amazonian and West Indian manatee species from Brazil
Source: Genet Mol Biol. 2021 Apr 9;44(2):e20190252. doi: 10.1590/1678-4685-GMB-2019-0252 (PMC8042642; doi:10.1590/1678-4685-GMB-2019-0252)
Supplement: Table S3 - [file 1415-4757-GMB-44-2-e20190252-s3.pdf]

Genetics and Molecular Biology
https://doi.org/10.1590/1678-4685-GMB-2019-0252

Supplementary Material to "TLR4 and TLR8 variability in Amazonian and West Indian manatee species from Brazil"

Table S3. Accession number of TLR4 and TLR8 sequences in Amazonian and West Indian manatees deposited in GenBank.

Code	Species	TLR4	TLR8
Tinu01	*Trichechus inunguis*	MN005119	MN005161
Tinu02	*Trichechus inunguis*	MN005120	MN005162
Tinu03	*Trichechus inunguis*	MN005121	MN005163
Tinu04	*Trichechus inunguis*	MN005122	MN005164
Tinu05	*Trichechus inunguis*	MN005123	MN005165
Tinu06	*Trichechus inunguis*	MN005124	MN005166
Tinu08	*Trichechus inunguis*	MN005125	MN005167
Tinu09	*Trichechus inunguis*	MN005126	MN005168
Tinu10	*Trichechus inunguis*	MN005127	MN005169
Tinu11	*Trichechus inunguis*	MN005128	MN005170
Tinu12	*Trichechus inunguis*	MN005129	MN005171
Tinu13	*Trichechus inunguis*	MN005130	MN005172
Tinu14	*Trichechus inunguis*	MN005131	MN005173
Tinu15	*Trichechus inunguis*	MN005132	MN005174
Tinu16	*Trichechus inunguis*	MN005133	MN005175
Tinu33	*Trichechus inunguis*	MN005134	MN005176
Tinu34	*Trichechus inunguis*	MN005135	MN005177
Tinu35	*Trichechus inunguis*	MN005136	MN005178
Tinu36	*Trichechus inunguis*	MN005137	MN005179
Tinu39	*Trichechus inunguis*	MN005138	MN005180
Tinu41	*Trichechus inunguis*	MN005139	MN005181
Tinu42	*Trichechus inunguis*	MN005140	MN005182
Tinu43	*Trichechus inunguis*	MN005141	MN005183
Tinu46	*Trichechus inunguis*	MN005142	MN005184
Tinu47	*Trichechus inunguis*	MN005143	MN005185
Tinu48	*Trichechus inunguis*	MN005144	MN005186
Tman18	*Trichechus manatus*	MN005145	MN005187
Tman19	*Trichechus manatus*	MN005146	MN005188
Tman20	*Trichechus manatus*	MN005147	MN005189
Tman21	*Trichechus manatus*	MN005148	MN005190
Tman23	*Trichechus manatus*	MN005149	MN005191
Tman24	*Trichechus manatus*	MN005150	MN005192
Tman25	*Trichechus manatus*	MN005151	MN005193

Code	Species	TLR4	TLR8
Tman26	*Trichechus manatus*	MN005152	MN005194
Tman27	*Trichechus manatus*	MN005153	MN005195
Tman28	*Trichechus manatus*	MN005154	MN005196
Tman29	*Trichechus manatus*	MN005155	MN005197
Tman30	*Trichechus manatus*	MN005156	MN005198
Tman31	*Trichechus manatus*	MN005157	MN005199
Tman32	*Trichechus manatus*	MN005158	MN005200
Tman37	*Trichechus manatus*	MN005159	MN005201
Tman44	*Trichechus manatus*		MN005202
Tman45	*Trichechus manatus*	MN005160	MN005203

Tman 45: Trichechus hybrid.
