# Supplementary material for: TLR4 and TLR8 variability in Amazonian and West Indian manatee species from Brazil
Source: Genet Mol Biol. 2021 Apr 9;44(2):e20190252. doi: 10.1590/1678-4685-GMB-2019-0252 (PMC8042642; doi:10.1590/1678-4685-GMB-2019-0252)
Supplement: Table S4 - [file 1415-4757-GMB-44-2-e20190252-s4.pdf]

Genetics and Molecular Biology
https://doi.org/10.1590/1678-4685-GMB-2019-0252

Supplementary Material to "TLR4 and TLR8 variability in Amazonian and West Indian manatee species from Brazil"

Table S4. Accession numbers of the TLR4 and TLR8 from GenBank.

Species		Accession number TLR4	TLR8
Aardvark	*Orycteropus afer afer*	XP_007934862.1	XP_007949818.1
Cape Golden mole	*Chrysochloris asiatica*	XP_006865788.1	XP_006835691.1
Elephant	*Loxodonta africana*	XP_003407827.1	XP_003416037.1
Elephant shrew	*Elephantulus edwardii*	XP_006890760.1	XP_006892434.1
Florida manatee	*Trichechus manatus*	XP_004372178.2<br>XP_012409812.1	XP_004386403.1
Tenrec	*Echinops telfairi*	XP_004712642.1	XP_004710022.1
Bovine	*Bos taurus*	NP_776623.5	NP_001029109.1
Killer whale	*Orcinus orca*	AB492857	XP_004284963.1
Sperm whale	*Physeter catodon*	AB500181	XM_007121208.1
Baiji	*Lipotes vexillifer*	JN642614	XM_007455053.1
Beluga whale	*Delphinapterus leucas*	JN642610	XM_022559817.1
Bottlenose dolphin	*Tursiops truncatus*	JN642619	XM_019928532.1
Striped dolphin	*Stenella coeruleoalba*	JN642618	KY119249.1
Minke whale	*Balaenoptera acutorostrata*	JN642608	XM_007180332.1
Pacific white-sided dolphin	*Lagenorhynchus obliquidens*	BAJ19433.1	
Buffalo	*Bubalus bubalis*	XM_025278946.1	XM_006077059.2
Camel	*Camelus dromedarius*	XM_010998309.2	XM_010993656.2
Horse	*Equus caballus*	AY005808.1	XM_005613945.3
Pig	*Sus scrofa*	AY753179.1	XM_021079505.1
Sheep	*Ovis aries*	GU461886.1	XM_012106133.3
