# Supplementary material for: TLR4 and TLR8 variability in Amazonian and West Indian manatee species from Brazil
Source: Genet Mol Biol. 2021 Apr 9;44(2):e20190252. doi: 10.1590/1678-4685-GMB-2019-0252 (PMC8042642; doi:10.1590/1678-4685-GMB-2019-0252)
Supplement: Table S5 - [file 1415-4757-GMB-44-2-e20190252-s5.pdf]

Genetics and Molecular Biology

https://doi.org/10.1590/1678-4685-GMB-2019-0252

Supplementary Material to “TLR4 and TLR8 variability in Amazonian and West Indian manatee species from Brazil”

Table S5. Identification of SNPs for the TLR4 in Amazonian (*Trichechus inunguis*) and West Indian (*Trichechus manatus*) manatees.

Code	510bp	645bp	782bp	1,135bp	1,824bp	1,864bp	2,140bp
Tinu01	A	C	T	C	A	T	C
Tinu02	A	C	T	C	A	T	C
Tinu03	A	C	T	C	A	T	C
Tinu04	A	C	T	C	A	T	C
Tinu05	A	C	T	C	A	T	C
Tinu06	A	C	T	C	A	T	C
Tinu08	A	C	T	C	A	T	C
Tinu09	A	C	T	C	A	T	C
Tinu10	A	C	T	C	A	T	C
Tinu11	A	C	T	C	A	T	C
Tinu12	A	C	T	Y (T/C)	W (A/T)	T	C
Tinu13	A	C	T	C	A	T	C
Tinu14	A	C	T	C	A	T	C
Tinu15	R (A/G)	S (C/G)	Y (T/C)	C	A	W (A/T)	C
Tinu16	R (A/G)	S (C/G)	Y (T/C)	Y (T/C)	A	W (A/T)	C
Tinu33	A	C	T	C	A	T	C
Tinu34	A	C	T	C	A	T	C

Code	510bp	645bp	782bp	1,135bp	1,824bp	1,864bp	2,140bp
Tinu35	A	C	T	Y (T/C)	W (A/T)	T	C
Tinu36	A	C	T	C	A	T	C
Tinu39	A	C	T	C	W (A/T)	T	Y (T/C)
Tinu41	A	C	T	C	A	T	C
Tinu42	A	C	T	C	A	T	C
Tinu43	A	C	T	Y (T/C)	A	T	C
Tinu46	A	C	T	C	A	T	C
Tinu47	A	C	T	C	A	T	C
Tinu48	A	S (C/G)	T	C	A	T	C
Tman18	G	G	C	C	A	A	C
Tman19	R (A/G)	S (C/G)	Y (T/C)	C	A	W (A/T)	C
Tman20	G	G	C	C	A	A	C
Tman21	R (A/G)	S (C/G)	Y (T/C)	C	A	W (A/T)	C
Tman23	G	G	C	C	A	A	C
Tman24	R (A/G)	S (C/G)	Y (T/C)	C	A	W (A/T)	C
Tman25	A	C	T	C	A	T	C
Tman26	G	G	C	C	A	A	C
Tman27	G	G	C	C	A	A	C
Tman28	A	C	T	C	A	T	C
Tman29	G	G	C	C	A	A	C
Tman30	R (A/G)	S (C/G)	Y (T/C)	C	A	W (A/T)	C
Tman31	A	C	T	C	A	T	C
Tman32	G	G	C	C	A	A	C
Tman37	A	C	T	C	A	T	C
Tman44	A	C	T	C	A	T	C
Tman45	R (A/G)	S (C/G)	Y (T/C)	C	A	W (A/T)	C

Tinu: *Trichechus inunguis*; Tman: *Trichechus manatus; Tman 45: Trichechus hybrid.*
