# Supplementary material for: TLR4 and TLR8 variability in Amazonian and West Indian manatee species from Brazil
Source: Genet Mol Biol. 2021 Apr 9;44(2):e20190252. doi: 10.1590/1678-4685-GMB-2019-0252 (PMC8042642; doi:10.1590/1678-4685-GMB-2019-0252)
Supplement: Table S6 - [file 1415-4757-GMB-44-2-e20190252-s6.pdf]

Genetics and Molecular Biology
https://doi.org/10.1590/1678-4685-GMB-2019-0252

Supplementary Material to "TLR4 and TLR8 variability in Amazonian and West Indian manatee species from Brazil"

Table S6. Identification of SNPs for the TLR8 in Amazonian (*Trichechus inunguis*) and West Indian (*Trichechus manatus*) manatees.

Code	212bp	216bp	297bp	346bp	810bp	1,584bp	1,659bp	2,545bp
Tinu01	T	T	C	A	G	T	A	G
Tinu02	T	C	Y (C/T)	R (G/A)	G	T	R (G/A)	R (G/A)
Tinu03	T	C	T	G	G	T	G	N
Tinu04	T	C	Y (C/T)	R (G/A)	G	T	R (G/A)	R (G/A)
Tinu05	T	C	T	G	G	T	G	A
Tinu06	T	C	Y (C/T)	R (G/A)	G	T	N	R (G/A)
Tinu08	T	C	C	A	G	T	R (G/A)	G
Tinu09	T	C	Y (C/T)	R (G/A)	G	T	R (G/A)	R (G/A)
Tinu10	T	C	C	A	G	T	G	G
Tinu11	T	C	T	G	G	T	G	A
Tinu12	T	C	T	G	G	T	G	A
Tinu13	T	C	C	A	G	T	R (G/A)	G
Tinu14	T	C	T	G	G	T	N	G
Tinu15	T	C	C	A	G	T	N	R (G/A)
Tinu16	T	C	C	A	N	T	A	G
Tinu33	T	C	T	G	G	T	G	A
Tinu34	T	C	Y (C/T)	R (G/A)	G	T	G	R (G/A)
Tinu35	T	C	T	G	G	T	G	A
Tinu36	T	C	T	G	G	N	G	A
Tinu39	K (T/G)	C	Y (C/T)	R (G/A)	N	N	R (G/A)	R (G/A)
Tinu41	T	C	T	G	N	T	G	A

Code	212bp	216bp	297bp	346bp	810bp	1,584bp	1,659bp	2,545bp
Tinu42	T	C	T	G	G	T	G	A
Tinu43	T	C	T	G	G	T	G	A
Tinu46	T	C	Y (C/T)	R (G/A)	G	T	N	R (G/A)
Tinu47	T	C	T	G	G	T	N	R (G/A)
Tinu48	T	C	C	A	G	T	N	G
Tman18	T	C	T	G	G	C	G	G
Tman19	T	C	T	G	G	C	G	G
Tman20	T	C	T	G	G	N	N	G
Tman21	T	C	T	G	G	C	N	N
Tman23	T	C	T	G	G	C	N	G
Tman24	T	C	T	G	G	C	N	G
Tman25	T	C	T	G	G	C	N	N
Tman26	T	C	T	G	G	C	N	G
Tman27	T	C	T	G	G	C	G	G
Tman28	T	C	Y (C/T)	R (G/A)	R (G/A)	Y (C/T)	G	G
Tman29	T	C	T	G	G	C	G	G
Tman30	T	C	T	G	G	C	G	G
Tman31	T	C	Y (C/T)	G	G	Y (C/T)	G	G
Tman32	T	C	T	G	G	C	G	G
Tman37	T	C	T	G	G	C	G	G
Tman44	T	C	T	G	G	N	N	G
Tman45	T	C	C	G	G	T	N	G

Tinu: *Trichechus inunguis*; Tman: *Trichechus manatus. Tman 45: Trichechus hybrid;* N:due to the low quality of some sequences, "N" was inserted, and it was not possible to identify any SNP.
